# Emerging roles of epigenetic regulators during lung development

**DOI:** 10.1038/s41419-025-07823-6

**Published:** 2025-07-28

**Authors:** Guohua Meng, Wenli Yin, Yue Hong, Salvatore Oliviero

**Affiliations:** 1 School of Life and Health Sciences, Hainan Province Key Laboratory of One Health, Collaborative Innovation Center of Life and Health, Hainan University, Haikou, Hainan China; 2 Department of Life Sciences and Systems Biology, University of Turin, Torino, Italy; 3 IIGM Foundation, Italian Institute for Genomic Medicine, Candiolo, Torino Italy; 4 Candiolo Cancer Institute, FPO-IRCCS, Candiolo, Torino Italy

**Keywords:** DNA methylation, Epigenetic memory, Epigenetic memory, Predictive markers, Respiratory tract diseases

## Abstract

Epigenetic pathways, including DNA methylation, non-coding RNAs, histone modification, and chromatin remodeling, control spatiotemporal gene expression and tightly coordinate the activities during embryogenesis. Emerging evidence indicates that epigenetic regulators are critically required for the maintenance of normal lung development and that the epigenetic marks are altered in lung cells during disease progression. In this review, we focus on the recent studies that have yielded insights into how the levels and patterns of epigenetic regulators are changed, and how these regulators contribute to the regulation of various stages during lung development. A deeper understanding of these epigenetic mechanisms could offer novel therapeutic targets for preventing fetal lung diseases.

## Facts

- Epigenetic regulators modulate cell fate decisions and functional homeostasis for the maintenance of normal lung development.
- Epigenetic dysregulation significantly contributes to lung cell dysfunction and the progression of disease-related tissue injury.
- Elucidating the mechanisms of epigenetic regulation during normal lung development could provide valuable insights into the in vivo regulation of cellular processes and the establishment and maintenance of overall organismal health.

## Open questions

- Given the observed variabilities in methylation profiles across human populations, how could a deeper investigation into methylation patterns across diverse populations help uncover novel therapeutic targets?
- The mechanisms by which non-coding RNAs coordinate with other molecular factors to regulate lung cell fate remain largely unexplored.
- Can we develop new computational methods for the prediction of chromatin interaction and organization and find more chromatin-remodeling factors related to lung development process?
- How are microenvironmental cues linked to epigenetic alterations in fetal lung diseases?
- Can we manipulate epigenetic marks precisely in a spatiotemporal and locus-specific manner?

## The neglected role of epigenomics in lung development

The lung is one of the most intricate organs in the human body, originating from a rudimentary foregut outpouching and developing into a highly complex and finely structured organ with multiple specialized cell types essential for gas exchange, mucociliary clearance, immune defense, and other critical processes. The human lungs comprise various cell types, including epithelial cells, endothelial cells (both vascular and lymphatic), pleural/mesothelial cells, airway and vascular smooth muscle cells, pericytes, fibroblasts, neurons, and immune cells such as alveolar macrophages. Epithelial cells, which are in direct contact with inhaled air, exhibit regional specialization with multiple stem cell and progenitor populations organized along the proximal-distal axis. These populations include basal cells, club cells, bronchoalveolar stem cells, and alveolar type 2 epithelial cells [1–4].

Extensive research has elucidated the precise transcription factor cascades driving tracheal and branching morphogenesis, which are integral to constructing the epithelial components of the respiratory system. Notably, lineage tracing maps, initially developed in mice [5], have significantly advanced our understanding of the origins of all epithelial cells in the adult lung, marking a crucial milestone in characterizing lung development.

Despite these achievements, recent technological breakthroughs in the isolation, culture, and characterization of the lung epithelium are challenging established definitions of lung epithelial cell identities, forms, and functions [6–9]. Classical cell types such as basal, goblet, club, ciliated, and alveolar cells are being redefined through revolutionary single-cell profiling technologies, revealing specialized subtypes, intermediate cell states, and highly plastic disease-driven stem populations [10, 11]. The precise approaches conducted by numerous scientists have often overlooked these specialized, intermediate, and transitory cell states that play critical roles in determining cell fate, highlighting the need to reassess our current scientific understanding and technological approaches.

During both lung development and remodeling in response to disease or injury, gene expression must be precisely regulated to achieve the extensive phenotypic heterogeneity of cell types required for homeostasis and pathogenesis. Epigenetic mechanisms (Table 1), including DNA methylation, histone modifications, the action of non-coding RNAs (ncRNAs), and chromatin remodeling, play a crucial role in regulating information “beyond the genome” required for lung modeling and remodeling. Therefore, to fully understand lung composition and differentiation, it is essential to prioritize the study of the epigenomic landscape alongside transcription factors.

**Table 1:** Epigenetic regulators with known functions in lung development.

Action	Regulator	Mechanism (s)	Effect (s)	Refs
DNA methylation	Dnmt1	Promotes proper branching morphogenesis, maintains proximal endodermal cell fate, and suppresses premature activation of the distal epithelial fate	Maintain lung morphogenesis and epithelial fate specification	[17]
	Apaf-1	Proximal promoter methylation causes DNA damage-induced apoptosis	Maintain embryonic lung development	[19]
	TP53BP2	CpG island-related proximal promoter regions methylation inhibits embryonic morphogenesis	Maintain embryonic lung development	[19]
	VEGF-A	Promoter methylation of primary fetal distal lung epithelial cells plays a crucial role in the vascular growth of the cardiopulmonary system	Regulates lung development at pseudoglandular/canalicular stage	[20]
Non-coding RNA	miR-142-3p	Contributes to the proper proliferation of mesenchymal progenitors by controlling the level of WNT signaling	Inhibition of miR-142-3p leads to differentiation of mouse embryonic lung parbronchial smooth muscle cell progenitor cells	[26]
	miR302-367	Represses expression of the tumor suppressors Rbl2 and Cdkn1a	Promotes the proliferation of both proximal and distal lung progenitors but prevents their subsequent differentiation	[27]
	miR-326	Not evaluated	Regulates the expansion and normal branching pattern of the distal epithelium of the embryonic lung and plays an important role in the breakdown of interstitial integrity	[28]
	miR-17, miR-20a, miR-106b	Not evaluated	Downregulation of miR-17, miR-20a, and miR-106b caused significant branching defects in embryonic lung epithelial explants	[29]
	miR-449a	Increase the Mycn and Sox9 mRNA levels, and the Ki-67 and SOX9 protein levels	Stimulating distal epithelial progenitor proliferation and mucociliary differentiation	[30]
	miR-26a	Not evaluated	Inhibits the formation of dilated lumens and aerated regions and maturation of the alveolar structure	[31, 32]
	miR-127	Not evaluated	Regulates the number and size of terminal buds during lung development	[33]
	miR-17-92	Hdac3 regulates appropriate TGF-β signaling during pulmonary capsulation by inhibiting the expression of miR-17-92	Promotes alveolar type 1 cell spreading and lung sacculation	[35]
		Negatively correlated with promoter methylation and DNA methyltransferase expression	Contributes to the molecular pathogenesis of bronchopulmonary dysplasia	[38]
		Promotes proliferation of lung epithelial progenitor cells partly through repressing Rbl2 expression	Participates in early lung development and lung epithelial fate determination	[36]
	miR449/34	Inhibits the expression of Notch1 and Dll1 to repress ciliated cell differentiation	Facilitates multiciliogenesis and regulates postnatal maturation of airway epithelial cells	[39, 40]
	miR375	Upregulates β-catenin signaling	Regulates trans-differentiation of AEC2 into AEC1	[41]
	RP11-380D23.2	Downregulated by PARP1 binding to its genomic sequence, which in turn modulates the expression of PITX2	Influencing distal lung differentiation	[45]
	LL18/NANCI	Acts upstream of Nkx2.1 and downstream from Wnt signaling	Regulates lung endoderm gene expression	[46]
Histone modification	Hat1	Maintain cell proliferation and genomic stability	Promotes lung development	[51]
	Hdac1/2	Inhibits H3K9 acetylation at the Rb1 (cell-cycle inhibitor) promoter to regulate proper proliferation of early lung endoderm	Promote endoderm progenitor proliferation during lung development and airway regeneration	[53]
	HDAC3	Represses miR-17-92 expression to allow for proper TGF-β signaling during lung sacculation	Regulates lung alveolar epithelial cell remodeling	[35]
	Hopx	Interacts with Hdac2 to mediate repression of cardiac-specific genes	Regulates lung epithelial maturation	[55]
	Suv39H1 Suv39H2	Induces transcriptional silencing through histone H3 lysine 9 methylation, and directly repress the expression of Sftpa1 during hypoxia	Regulates fetal lung development	[56]
Chromatin remodeling	Jmjd3	Jmjd3-mediated alterations in gene expression (AQP-5, SP-B) are associated with locus-specific changes in the methylation status of H3K27 and H3K4	Promotes embryonic lung development	[58]
	MCRIP1	Interferes with interactions of CtBP with the lung-enriched transcriptional repressors, Foxp1 and Foxp2, thereby preventing the recruitment of the CtBP co-repressor complex to the SP-B and SP-C promoters and maintaining them in an active chromatin state	Maintains fatal respiratory function	[59]
	Ezh2	Represses Trp63 expression in lung epithelial development; Promotes smooth muscle differentiation from the mesothelium through activation of myocardin and Tbx18	Maintains normal lung endoderm development	[60, 61]
		Represses Igf1 expression and prevents basal cell differentiation in the developing lung	Promotes airway lineage specification and alveolarization	[62]

This review explores how epigenetic regulators control lung cell plasticity, emphasizing their functional adaptability. To provide clarity on this intricate subject, we focus on key epigenetic mechanisms, highlighting their expression and roles in the context of lung development. Understanding their contributions during development will offer deeper insights into how the epigenome orchestrates regulatory processes, particularly in relation to disease progression.

## Epigenetic marks and modifications during normal lung development

**Fig. 1:**
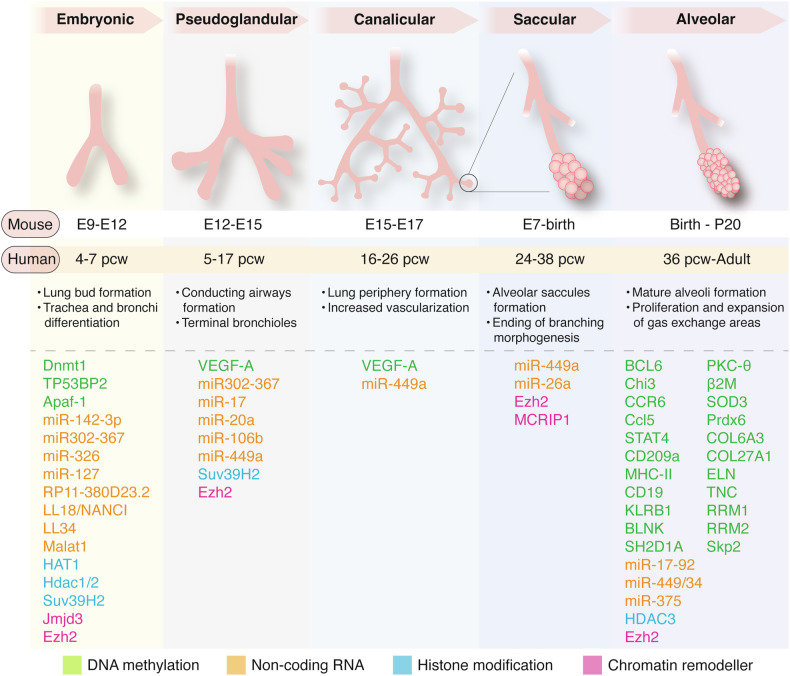
Epigenetic regulators involved in different stages of normal lung development. Lung development progresses through five sequential stages characterized by distinct anatomical and histological features: the embryonic, pseudoglandular, canalicular, saccular, and alveolar stages. During the embryonic and pseudoglandular stages, the conducting airways are established, whereas the gas-exchange regions, marked by increased vascularization and mesenchymal thinning, emerge during the canalicular, saccular, and alveolar stages. Developmental timeframes are specified in post-conception weeks (pcw) for humans and embryonic days (E) for mice. Circular area is enlarged to show the gas exchange occurring across the alveolar epithelium. Here, we provide an overview of epigenetic regulators involved at each developmental stage, categorized into DNA methylation (green), non-coding RNAs (orange), histone modifications (blue), and chromatin remodelers (pink).

Research characterizing the epigenomic alterations during normal lung development can be roughly divided into three categories. The first category focuses on the regulators and targets of CpG DNA methylation. The second category examines the role of ncRNAs in lung development. The third category investigates chromatin remodeling complexes at specific loci critical for lung function and the alterations in histone modifications or chromosomal architecture during differentiation. The various stages of lung development, along with the characteristics of each stage, are illustrated in Fig. 1. Additionally, we have systematically summarized the currently known epigenetic regulatory factors that function at different stages of lung development (Fig. 1, Table 1).

### DNA methylation

DNA methylation is a reversible DNA modification characterized by the addition of a methyl group to cytosine residues within nucleic acids. This modification is facilitated by DNA methyltransferase enzymes, specifically DNA methyltransferase 1 (DNMT1) for maintenance methylation, and DNMT3A and DNMT3B for de novo methylation. Active DNA demethylation is mediated by ten-eleven translocation (TET) enzymes, including TET1, TET2, and TET3. DNA methylation plays a pivotal role in the epigenetic regulation of mammalian embryonic development and is essential for numerous biological processes, including repression of gene transcription, maintenance of gene imprinting, X-chromosome inactivation, and suppression of transposable elements [12–16].

#### Embryonic stage

The embryonic stage is crucial for lung development, establishing the lung primordium (respiratory diverticulum) and initial branching of the bronchial tree. This phase lays the foundation for airway and vascular structures, with disruptions leading to severe malformations like pulmonary hypoplasia or tracheoesophageal fistula. This stage encompasses ~4–7 post-conception weeks (pcw). During this period, the primary left and right lung buds emerge from the foregut endoderm around the end of week 4, subsequently undergoing rapid branching to establish the fundamental lobular structure of the lung by the end of week 5 (Fig. 1). DNMT1 functions as both a de novo and maintenance methyltransferase, which is essential for embryonic development. This is evidenced by Dnmt1 null mice experiencing developmental arrest by embryonic day 9.5 (E9.5) and resulting in lethality before embryonic day 11.0 (E11.0) (Table 1) [16]. Dnmt1 maintains DNA methylation patterns on the newly synthesized strand during DNA replication and is essential for the spatiotemporal regulation of lung branching morphogenesis in mice [17], while TET2, which is highly expressed during the late stages of murine lung development [18], may act as its opposition, suggesting that DNA methylation dynamics play a critical role in the transcriptional regulation underlying lung parenchyma formation.

**Fig. 2:**
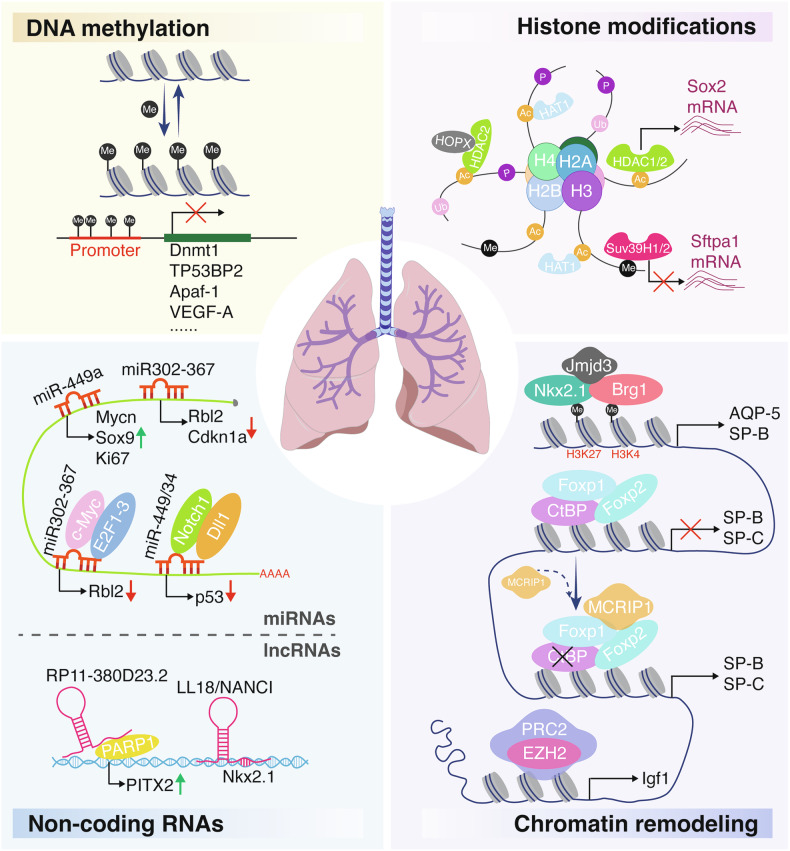
Epigenetic regulation mechanisms related to lung development. The epigenetic landscape guiding lung development is likely regulated through dynamic changes in DNA methylation, histone modifications, non-coding RNAs, and chromatin remodeling. These epigenetic modifications facilitate transcriptional shifts essential for key developmental processes in the lung. Abbreviations: Ac acetylation, Me methylation, P phosphorylation, Ub ubiquitination, H2A, H2B, H3, and H4, core histones.

In human embryonic lung cells, methylation of CpG islands has been frequently observed in the proximal promoter regions of TP53BP2 (tumor protein p53 binding protein 2) and Apaf-1 (apoptotic protease activating factor-1) (Fig. 2) [19]. Notably, inhibition of methylation significantly upregulated Apaf-1 expression in these cells. Furthermore, the CpG island-associated proximal promoter regions of Apaf-1 and TP53BP2 are transcription factor binding sites. Therefore, the methylation of Apaf-1 and TP53BP2 may impede embryonic morphogenesis and play a crucial role in early lung development.

#### Pseudoglandular/canalicular/saccular stage

The pseudoglandular phase of lung development occurs from ~5 to 17 pcw. During this time, the lung undergoes extensive branching morphogenesis, establishing the airway tree, which begins to differentiate with the formation of cartilage, smooth muscle, and mucous glands (Fig. 1). The subsequent canalicular phase spans 16–26 pcw, characterized by an estimated three additional rounds of epithelial branching to develop the future alveolar regions (Fig. 1). The saccular stage, extending from ~24 to 38 pcw, marks the conclusion of branching morphogenesis. During this stage, the distal airspaces evolve into thin-walled terminal saccules, clustering at the ends of the airways (Fig. 1).

During the pseudoglandular and canalicular stages of lung development, CpG island methylation in the VEGF-A promoter of primary fetal distal lung epithelial cells is critical for the vascular growth of the cardiopulmonary system (Fig. 2) [20]. Moreover, a recent study demonstrated that Dnmt1 is essential for early branching morphogenesis of the lungs and the regulation of epithelial fate specification [17]. The absence of Dnmt1 results in early branching defects, loss of epithelial polarity, and improper differentiation of proximal endodermal cells, coupled with an expansion of the distal endoderm compartment. Dnmt1 deficiency also disrupts epithelial-mesenchymal interactions, leading to premature differentiation of distal endodermal cells and the early expression of genes specific to alveolar type 2 cells [17]. This data reveals an important requirement for Dnmt1 mediated DNA methylation in early lung development to promote proper branching morphogenesis, maintain proximal endodermal cell fate, and suppress premature activation of the distal epithelial fate. In conclusion, these studies suggest an important role for DNA methylation in the correct temporal activation of specific transcriptional programs during lung development.

#### Alveolar stage

The alveolar stage is crucial for alveolar formation, where septae grow from the saccular walls, subdividing distal saccules into alveoli and thereby increasing the surface area for gas exchange. Traditionally, alveolar formation has been thought to occur from 36 weeks gestation to 3 years postnatally (Fig. 1). However, advanced imaging technologies and detailed stereological analyses suggest that alveolar formation extends into young adulthood (~21 years) [21, 22].

During alveolar septation, DNA methylation levels are altered around key factors known to be involved in lung differentiation, such as Wnt family members and SOX9 [23]. The statistical integration of DNA methylation, proteomic, gene and miRNA expression changes during alveolar maturation [24] has revealed key signaling hubs involving known transcription factors such as NKX2-1, GLI and CEBPA, as well as identifying previously uncharacterized roles for POU2F1 and TMEM37, which plays a role in multiple lung diseases.

In addition, recent studies have also identified DNA methylation-regulated genes critical for normal alveolar septation [23]. Key players include inflammatory mediators (e.g., BCL6, STAT4, CCL5), antioxidant defenses (SOD3, Prdx6), and extracellular matrix regulators (COL6A3, ELN), alongside oncogenes linked to lung cancer (RRM1, RRM2). Furthermore, differentially methylated CpGs at birth have been mapped, revealing epigenetic signatures of fetal lung development (Fig. 1) [25]. These findings underscore the role of DNA methylation in orchestrating both physiological and pathological lung maturation.

### Non-coding RNAs

ncRNAs, such as microRNAs (miRNAs) and long noncoding RNAs (lncRNAs), play a crucial role in the epigenetic regulation of lung development.

#### MicroRNAs

miRNAs are a class of small noncoding RNA which exert post-transcriptional gene regulation activity by targeting messenger RNAs. In recent years, miRNAs have rapidly emerged not only as critically contributing to various pulmonary diseases but also to normal development of the lung and maintenance of its homeostasis. The evidence that miRNAs play a role in lung development derive from mice with conditional knockout of Dicer in lung epithelial cells that exhibit a failure of epithelial branching, underscoring the essential regulatory role of miRNAs in lung epithelial morphogenesis. Further, evaluation of miRNA expression patterns during the different stages of lung development revealed a number of miRNAs which are differentially expressed.

At the embryonic stage, several miRNAs have been reported to regulate the maintenance of lung epithelium and mesenchyme. For instance, miR-142-3p is notably upregulated in the embryonic lung mesenchyme (Fig. 1), where it restricts the ectopic differentiation of parabronchial smooth muscle cell progenitors (Table 1) [26]. Concurrently, another miRNA, miR-302-367 cluster, exerting their functions by regulating the balance between lung epithelial proliferation and differentiation. This miRNA cluster predominantly expressed in the embryonic lung epithelium before embryonic day 15 (Fig. 1), and promotes the proliferation of both proximal and distal lung progenitors by directly repressing the tumor suppressors Rbl2 and Cdkn1a (Fig. 2) [27]. Another miRNA that plays a pivotal function in embryonic lung development is miR-326. This miRNA is essential for the expansion of the distal epithelium and maintaining regular branching patterns and mesenchymal integrity in the embryonic lung [28].

As development progresses, other miRNAs exert their functions by regulating branching and epithelial fate at mid-stage lung development (includes pseudoglandular, canalicular, and saccular stages). For instance, miR-17 and its paralogs, miR-20a and miR-106b, are highly expressed in the lung (Fig. 1). Downregulation of these miRNAs leads to significant branching defects in embryonic lung epithelial explants [29]. Another example is miR-449a, which peaks during mid-gestation but declining postnatally (Fig. 1). This miRNA emerges as a key modulator of distal epithelial proliferation and mucociliary differentiation via upregulation of Mycn, Sox9, and Ki-67 (Fig. 2) [30]. Aside from guiding the branching and epithelial fate determination, miRNAs also regulate lung structural maturation. miR-26a is highly expressed in the rat fetal lung, especially during the saccular stage (Fig. 1) [31]. This miRNA ensures proper lumen formation and alveolar maturation; its knockout leads to dilated airways and disrupted alveolarization [32]. Besides, miR-127 is highly expressed during late fetal lung development and fine-tunes terminal bud size and number, facilitating late-stage patterning (Fig. 1) [33].

At the alveolar stage, the maintenance of alveolar epithelial stem cell function relies on a tightly regulated genetic network, with emerging evidence identifying miRNAs as essential components of this regulatory program. One of the best examples of this subset of miRNAs is miR-17-92, which was the first group of miRNAs to be implicated in a developmental syndrome in humans. In bronchopulmonary dysplasia (BPD), miR-17-92 levels are markedly reduced, correlating with increased promoter methylation and elevated DNA methyltransferase activity [34]. Moreover, the miR-17-92 cluster promotes alveolar type 1 cells expansion and lung maturation [35]. Functionally, miR-17-92 maintains lung epithelial progenitor cell proliferation and an undifferentiated state, mediated in part through repression of the cell cycle inhibitor Rbl2 [36]. This cluster is transcriptionally activated by the oncoproteins c-Myc and E2F1-3 (Fig. 2) [37], underscoring its importance in cellular proliferation. Consistent with its essential role, miR-17-92 knockout mice exhibit lethal lung hypoplasia postnatally [38].

In the later stages of lung development, there is a crucial transition from proliferation to differentiation, which is supported by changes in the expression and function of various miRNAs. For instance, miR-449/34 is highly expressed during late development and suppresses proliferation by promoting p53 (Trp53) acetylation and activation. This miRNA cluster also facilitates multiciliogenesis and is further upregulated following birth or exposure to air, indicating its potential role in the postnatal maturation of airway epithelial cells. The regulation of multiciliogenesis by miR-449/34 is partly mediated through the inhibition of Notch1 and Dll1, both of which repress ciliated cell differentiation (Figs. 1 and 2) [39, 40]. Another significant miRNA in the late stages of lung development is miR-375, which is upregulated just before birth in alveolar epithelial type II cells (AEC2) and remains highly expressed postnatally (Fig. 1). When AEC2 cells are cultured on plastic plates, the levels of miR-375 decrease, leading to the trans-differentiation of AEC2 into alveolar epithelial type I (AEC1) cells via the upregulation of β-catenin signaling [41]. A similar trans-differentiation process occurs during lung development, although the precise role of miR-375 in regulating this process in vivo through the inhibition of β-catenin signaling remains unclear.

#### Long noncoding RNAs

Numerous lncRNAs are expressed in the lung, many of which also regulate developmental processes like differentiation and proliferation in other tissues [42–44]; They usually act through various mechanisms and interactions with proteins, DNA, and RNA. however, direct evidence linking lncRNAs to lung development is limited. LncRNA RP11-380D23.2 has been identified as a cis-regulator of its nearest gene PITX2, which plays a crucial role in distal lung morphogenesis via WNT signaling pathway (Table 1). The expression of RP11-380D23.2 is downregulated by PARP1 binding to its genomic sequence, which in turn modulates the expression of PITX2, influencing distal lung differentiation (Figs. 1 and 2) [45].

Moreover, previous study showed that two lncRNAs, Nkx2.1-associated noncoding intergenic RNA (LL18/NANCI) and LL34, play distinct roles in endoderm development by controlling expression of critical developmental transcription factors and pathways, including retinoic acid signaling. In particular, LL18/NANCI acts upstream of Nkx2.1 and downstream from Wnt signaling to regulate lung endoderm gene expression (Fig. 2) [46]. These studies reveal that lncRNAs play an important role in foregut and lung endoderm development by regulating multiple aspects of gene transcription, often through regulation of transcription factor expression.

Another lncRNA that plays a function in lung development is metastasis-associated lung adenocarcinoma transcript 1 (MALAT1). MALAT1 is highly expressed in the developing lung and was initially suggested to promote lung cancer proliferation and metastasis. Although the loss of MALAT1 does not affect lung development, a previous study indicated that the upregulation of MALAT1 may protect preterm infants with bronchopulmonary dysplasia by inhibiting apoptosis [47–49].

### Histone modifications

Recent studies have also highlighted epigenetic mechanisms, such as histone modifications, as crucial regulators of lung development. Histones undergo various modifications, including methylation, acetylation, phosphorylation, and ubiquitylation, at specific amino acids along the histone tail. Among these, histone acetylation plays a significant role in regulating lung development and function. Histone acetyltransferases (HATs) facilitate the acetylation of histone tails, promoting gene transcription, while histone deacetylases (HDACs) remove acetyl groups, leading to gene silencing. Although HATs and HDACs are primarily studied for their histone-modifying capabilities, they also target a wide range of proteins, including other epigenetic regulators and transcription factors [50]. This ability to modify various proteins broadens the potential influence of HATs and HDACs and complicates the interpretation of their roles in lung development.

During embryonic lung development, a precise balance between the activities of HDACs and HATs is crucial. HAT1 is essential for the acetylation of newly synthesized histones H3 and H4 (Fig. 2). In Hat1^-/-^ mice, embryonic fibroblasts exhibit high levels of genomic instability and increased sensitivity to DNA disruptors. Furthermore, HAT-1 deficiency can lead to defects in lung development, resulting in neonatal mortality (Table 1) [51].

In the lung epithelium, the loss of HDAC1 and HDAC2 reduces Sox2 expression and impairs the development of multiple proximal cell types (Fig. 2) [52]. This alteration in Sox2 expression is partly mediated by increased Bmp4 expression, a direct target of HDAC1 and HDAC2, contributing to the severe branching defects observed in HDAC1/2 mutants. HDAC1 and HDAC2 also promote endoderm progenitor proliferation during lung development and airway regeneration by repressing the tumor suppressors retinoblastoma 1 (Rb1), p16 (Cdkn2a) and p21 (Cdkn1a) [53].

Moreover, hyperoxia during neonatal development has been shown to decrease HDAC1/2 activity, leading to alveolar hyperplasia and disrupted alveolarization [54]. Histone deacetylase 3 (HDAC3) also plays a critical role in regulating the spreading of alveolar type 1 (AT1) cells, which is necessary for distal alveolar maturation [35].

In addition to their roles as histone deacetylases, HDACs may influence lung development through interactions with non-histone proteins. Recent data indicate that Hopx, a transcriptional repressor, does not directly bind DNA but instead interacts with HDAC2 (Fig. 2). Hopx is broadly expressed in the developing lung epithelium after embryonic day 13.5 (E13.5). Approximately 25% of Hopx-null animals die within 24 h of birth due to increased surfactant expression and disrupted alveolarization [55]. These findings suggest that Hopx-HDAC complexes play a crucial role in regulating lung epithelial maturation, potentially offering a new therapeutic target for enhancing epithelial maturation in preterm infants or following lung injury.

While histone acetylation is known to significantly impact lung development, the roles of other epigenetic complexes remain less understood. The methyltransferases Suv39H1 and Suv39H2, which induce transcriptional silencing through histone H3 lysine 9 methylation, have been shown to directly repress the expression of the surfactant protein SP-A (Sftpa1) during hypoxia (Figs. 1 and 2) [56]. These methyltransferases are also highly expressed during early lung development, suggesting that they inhibit SP-A transcription until later stages of development.

### Chromatin remodeling

Mechanistic studies have demonstrated that proper lung development and differentiation necessitate the temporally and spatially coordinated deposition of various histone marks by chromatin-remodeling complexes and factors modulating their activity, such as Jumonji domain containing-3 (JMJD3), MCRIP1 and the EZH2-containing Polycomb Repressive Complex (PRC) (Fig. 1).

JMJD3 is a histone demethylase that specifically catalyzes the removal of repressive trimethylation marks from histone H3 lysine 27 (H3K27me3), converting it to H3K27me1/me2 or unmethylated H3K27 [57]. This enzymatic activity antagonizes PRC2-mediated gene silencing, enabling dynamic transcriptional activation. By erasing H3K27me3, JMJD3 facilitates an open chromatin state, permitting access for transcription factors and RNA polymerase II to promoters of developmentally critical genes. Global deletion of Jmjd3 in mice leads to perinatal lethality due to defective lung development. However, tissue and stage-specific deletions have shown that Jmjd3 is dispensable in the later stages of embryonic lung development. Ablation of Jmjd3 results in the downregulation of genes essential for lung development and function, including AQP-5 and SP-B. These Jmjd3-mediated gene expression alterations are associated with locus-specific changes in the methylation status of H3K27 and H3K4. Additionally, Jmjd3 is recruited to the SP-B promoter through interactions with the transcription factor Nkx2.1 and the epigenetic protein Brg1 (Fig. 2 and Table 1) [58]. JMJD3 exemplifies how histone demethylases serve as “epigenetic switches,” bridging chromatin dynamics with gene regulatory networks in lung development and disease.

MCRIP1 is a scaffold protein that interacts with chromatin remodelers, transcription factors, and non-coding RNAs to regulate gene expression. While not a canonical chromatin remodeler itself, it plays a critical adapter role in facilitating chromatin dynamics.

Recently, MCRIP1 has been reported to act as a regulator of the CtBP transcriptional co-repressor to enhance the expression of surfactant proteins SP-B and SP-C by inhibiting CtBP-mediated epigenetic gene silencing [59]. Mice with homozygous deficiency of MCRIP1 suffer fatal respiratory distress due to abnormal transcriptional repression of these surfactant proteins. Subsequent findings indicate that MCRIP1 disrupts the interactions between CtBP and the lung-enriched transcriptional repressors Foxp1 and Foxp2. This disruption prevents the recruitment of the CtBP co-repressor complex to the SP-B and SP-C promoters, thereby maintaining these promoters in an active chromatin state (Fig. 2).

Furthermore, EZH2 is the catalytic subunit of the PRC2, which mediates gene silencing by depositing the repressive histone mark H3K27me3. This modification compacts chromatin, restricting access to transcription factors and RNA polymerase II. Ezh2 is critical for the development of both epithelial and mesenchymal lineages. Ezh2 represses the basal cell lineage fate in the epithelium and constrains the smooth muscle cell fate in the mesoderm [60, 61]. Moreover, Ezh2 is essential for airway lineage specification and alveolarization, with its loss in the lung epithelium resulting in defective lung formation and perinatal mortality [62]. Remarkably, RNA-seq analysis revealed that loss of Ezh2 de-represses insulin-like growth factor 1 (Igf1) expression, and modulation of IGF1 signaling ex vivo in wild-type lungs can induce basal cell differentiation (Fig. 2).

## Epigenetic alterations associated with fetal lung diseases

Emerging evidence highlights the existence of epigenetic memory during early lung development, which may be responsive to fetal lung diseases or neonatal lung injuries. To elucidate the role of epigenetics in the developmental origins of lung disease, it is crucial to identify molecular targets involved in both normal lung development and lung injury. Research should focus on characterizing the epigenetic states of these target genes and the regulators influencing these states.

Recent studies indicate that perinatal insults can alter gene expression and epigenetic determinants in the lung, as seen in intrauterine growth restriction (IUGR) models. In rat lungs, IUGR induces epigenetic modifications to the PPARγ gene, a nuclear receptor transcription factor essential for epithelial-mesenchymal interactions in lung development [63]. PPARγ is essential for various stages of lung development, including the differentiation of epithelial cells and the establishment of lung homeostasis [64]. Additionally, PPARγ is prominently expressed in the airway epithelium, contributing to normal lung maturation and function. Its signaling pathways are vital for the evolution and maintenance of lung physiology [65].

PPARγ is regulated epigenetically for tissue-specific and developmental transcription. IUGR decreases mRNA transcript levels of all PPARγ variants in neonatal rat lungs, associated with sex-specific alterations in H3 and H4 methylation [66, 67]. Specifically, IUGR reduces H3K9 trimethylation (H3K9me3) in male neonatal rats while increasing it in females, while control rats show no sex-specific differences in basal epigenetic regulation of PPARγ [67]. Interestingly, these H3K9me3 changes are linked to methyl CpG binding protein 2 (MeCP2), a regulator of H3K9me3. MeCP2 binds to PPARγ promoters, repressing transcription by increasing H3K9me3 [68]. IUGR increases MeCP2 expression and occupancy at PPARγ promoters in female neonatal lungs, but not in males [67]. This sex-specific epigenetic response to IUGR highlights an important area for ongoing research. Gender-divergence in molecular regulation during lung development is consistent with previous observations of subtle differences in the timing of lung maturation between males and females [69]. Epigenetic priming may contribute, as female lungs exhibit greater mass-specific surface area post-puberty, adapting to pregnancy-related oxygen demands [70]. It is possible that the epigenetic regulatory mechanisms governing MeCP2 expression and function may exhibit subtle differences in male and female lungs under basal conditions. However, under pathological stress such as IUGR, these differences become more pronounced resulting in a gender-divergence in MeCP2 response. This response may predispose to gender-specific differences in the severity and timing or lung morbidities.

Immune responses also play a crucial role in lung disease development, with epigenetic mechanisms implicated. In humans, asthma origins have been linked to maternal folate intake during pregnancy [71], which leads to the production of S-adenosyl-L-methionine, a universal methyl donor and precursor for DNA methylation. A high methyl donor diet during gestation increases airway inflammation, serum IgE levels, and airway hyperresponsiveness in mice [72]. The Runx3 gene, hypermethylated in mice on a high methyl donor diet, may mediate these responses [72]. Additionally, epigenetic control of Th1/Th2 lineage differentiation likely contributes to the allergic airway disease development [73, 74].

Moreover, several non-coding RNAs associated with fetal lung disease have also been identified. In the context of BPD, the expressions of hsa-miR-103a-3p and hsa-miR-185-5p are significantly decreased. In normal human umbilical vein endothelial cells, overexpression of hsa-miR-103a-3p and hsa-miR-185-5p significantly enhances endothelial cell proliferation, tubule formation, and cell migration. Conversely, overexpression of hsa-miR-200a-3p inhibits these cellular responses [75]. Additionally, miR-374a and miR-210 expression levels increase during hypoxia and ischemia in newborn piglets, suggesting their importance in regulating neonatal hypoxia and potential as biomarkers [76].

## Organoids systems for modeling human lung development and disease

Organoids, three-dimensional culture systems derived from stem cells, closely mimic the structural, biological, and functional characteristics of their organ of origin [77, 78]. In particular, lung organoid platforms have become powerful tools for modeling lung physiology and disease. There are three main types of lung organoids based on stem cell sources: adult lung stem/progenitor stem cells (AdSCs), fetal lung stem cells (FSCs), and induced pluripotent stem cells (iPSCs). These sources, combined with optimized culture conditions, such as specific media supplements and co-cultures with relevant cell types, facilitate the derivation and maintenance of various cell types.

AdSC-derived lung organoids are valuable for investigating epithelial stem cell potential and cellular interactions during both homeostasis and disease. Depending on the stem cell isolation site, human basal cells can form organoids like tracheospheres, bronchospheres, or nasospheres, comprising basal, club, ciliated, and goblet cells [79–81]. Efforts to extend the lifespan and differentiation potential of organoids from patient stem cells have also been successful. For instance, Sachs et al. developed a long-term human airway organoid model from human bronchoalveolar lavage and resection material. By modulating TGF-β, FGF, and WNT signaling pathways, these organoids maintained basal, ciliated, and secretory/club cells for over a year [82]. Moreover, mouse tracheospheres serve as a valuable platform for high-throughput screening of compounds that modulate basal cell proliferation and differentiation. This approach holds therapeutic potential for lung diseases characterized by altered ciliated-to-secretory cell ratios, including asthma, COPD, and cystic fibrosis - all associated with chronic inflammation and goblet cell hyperplasia [83]. Organoid-based systems enable systematic identification of small molecules capable of restoring epithelial homeostasis by directing basal cell fate decisions toward ciliated rather than secretory lineages, offering new avenues for drug discovery in airway pathologies.

FSC-derived lung organoids have also been instrumental in studying lineage specification and cellular interactions during development. In 2017, Nikolić et al. developed organoids from human and mouse fetal lung bud tips (LBTs) using a culture medium containing seven factors known to modulate critical lung morphogenesis signaling pathways [84]. This combination allowed the organoids to retain the expression of lung-specific transcription factors and markers without differentiating into mature cell types. Recently, Lim et al. have functionally validated cell-cell interactions in the developing human alveolar niche using human fetal lung tip progenitor cells-derived organoids system, showing that Wnt signaling from differentiating fibroblasts promotes alveolar-type-2 cell identity. This study also showed that differential binding of NKX2.1 coordinates alveolar maturation, allowing future study to model the effects of human genetic variation in NKX2.1 on alveolar differentiation [85]. This organoid system recapitulates key aspects of human fetal lung stem cell biology allowing mechanistic experiments to determine the cellular and molecular regulation of human development and disease.

The development of protocols for generating human iPSC-derived lung organoids marks a significant advancement in pulmonary disease modeling, drug screening, and regenerative medicine. These organoids, derived from definitive endoderm, sequentially generate anterior foregut endoderm, ventral anterior foregut endoderm cells (VAFECs), and, ultimately, NKX2-1^+^ lung progenitors [86–88]. A coculture system demonstrated that the CPM^+^ surface marker could isolate lung progenitors from VAFECs, which included AT1 and AT2 cells [89]. Current iPSC models mainly feature epithelial cells from lung alveolar or airway compartments, with few demonstrating proximal-to-distal patterning. For example, SOX9^+^SOX2^+^ lung bud organoids (LBOs) were generated to mimic the cellular composition of LBTs [90]. Although these LBOs lacked mature airway or AT1 cells, they contained goblet and club cells in the proximal structures and AT2 cells in the distal tips. Additionally, human lung organoids and bud tip organoids from iPSC-derived foregut spheroids were developed [91], showcasing airway-like epithelium surrounded by mesenchymal cells and epithelial cells coexpressing alveolar markers. Moreover, cystic fibrosis (CF) iPSC-derived lung organoids could provide a reliable and reproducible source of CF mutant cells for screening drugs that compensate for, or correct, patient-specific mutations [92]. This would overcome the problem of variability in the behavior of primary lung progenitor cells derived from even healthy individuals. Further optimization of culture conditions is essential to fully recapitulate lung architecture in iPSC-derived organoids.

## Conclusions and perspectives

The epigenetic landscape of lung development represents a dynamic and accurately orchestrated process, where regulators fine-tune gene expression with spatiotemporal precision. Understanding this process is pivotal for deciphering the developmental mystery and offering unprecedented therapeutic opportunities for lung disease diagnosis and treatment.

Epigenetic regulation is crucial during multiple stages of lung development, influencing lung stem cell self-renewal, fate determination, and functional homeostasis in vivo. Epigenetic marks and regulators have also been implicated in lung developmental disorders under specific conditions. Nevertheless, some key issues remain to be investigated in this field, including the identification of specific epigenetic signatures during normal human lung development, as well as targeting specific aberrant epigenetic marks in translational studies.

Although limited, existing epigenomic studies have begun to illuminate the mechanisms and factors governing normal lung development and differentiation. These studies have shifted our understanding and highlighted the potential for significant advances in the field. Recent technological developments, such as single-cell epigenomic profiling [7, 93–95], immortalization of lung epithelial cells [96], organoids generation systems [91, 96, 97], and targeted epigenome editing [98–100], offering promising tools for further elucidating the complexity of epigenomic actions in human lung development and disease.
